# The Telemedicine Program Design Canvas: a visual tool for planning telemedicine interventions

**DOI:** 10.1093/oodh/oqac002

**Published:** 2022-12-20

**Authors:** Neha Verma, Izabella Samuel, Samuel Weinreb, Mackenzie Hall, Kai Zhang, Mariana Bendavit, Vibha Bhirud, Jordan Shuff, Youseph Yazdi, Soumyadipta Acharya

**Affiliations:** Division of Biomedical Informatics & Data Science, Johns Hopkins University School of Medicine, Baltimore, MD, USA; Center for Bioengineering Innovation & Design, Johns Hopkins University, Baltimore, MD, USA; Center for Bioengineering Innovation & Design, Johns Hopkins University, Baltimore, MD, USA; Center for Bioengineering Innovation & Design, Johns Hopkins University, Baltimore, MD, USA; Center for Bioengineering Innovation & Design, Johns Hopkins University, Baltimore, MD, USA; Intelehealth, Mumbai, Maharashtra, India; Center for Bioengineering Innovation & Design, Johns Hopkins University, Baltimore, MD, USA; Center for Bioengineering Innovation & Design, Johns Hopkins University, Baltimore, MD, USA; Center for Bioengineering Innovation & Design, Johns Hopkins University, Baltimore, MD, USA

**Keywords:** project implementation, project planning, program design, digital health, telehealth, telemedicine

## Abstract

Telemedicine has seen widespread adoption during the COVID-19 pandemic. The implementation of telemedicine projects can be complex, with over 75% of telemedicine initiatives failing in the implementation phase. Health organizations that want to adopt telemedicine as part of their healthcare delivery programs struggle to plan and implement sustainable and scalable initiatives effectively. This paper presents the Telemedicine Program Design Canvas—a tool to guide health organizations in planning telemedicine interventions and drive intervention success. It was developed and validated through six workshops with users and stakeholders of telemedicine. Based on the workshops and the lessons learned from the subsequent interventions of these projects, we identified the 14 key elements that must be addressed while planning and implementing a telemedicine project. We organized these into a simple visual tool that health organizations could use. The 14 elements include the problem, ecosystem, patients, patient journey, patient engagement and trust, providers, provider training, provider engagement, channels, technology, medicines and diagnostics, desired outcomes, costs and revenues. The tool was then tested and validated by applying it with a new group of six telemedicine projects. Overall, the perspectives of 108 users and stakeholders of telemedicine projects, including organizational leadership, doctors, nurses, midwives, community health workers, patients, policymakers, technologists, legal and finance experts, were included in the development of the tool. The Telemedicine Program Design Canvas provides a structured and straightforward method for the rapid prototyping and holistic planning of telemedicine interventions.

## INTRODUCTION

In low- and middle-income countries (LMICs) with insufficient resources and infrastructure to adequately provide healthcare to every individual, telemedicine has become an increasingly common approach to expand access to treatment [1]. During the COVID-19 pandemic, the need for physical distancing required a rapid shift from in-person to remote care, galvanizing governments, health systems and organizations to invest in mobile health programs and technologies and accelerating the adoption of telemedicine [2]. Thus, telemedicine has been implemented to give patients in remote or low-resource settings access to primary care [3], specialist care, telesurgery, telementoring, chronic disease management and prevention [4, 5], health education [6] and medical services in disasters and other emergencies [7]. However, despite these telemedicine programs' large volume and breadth, there are still many barriers to successful implementation. It is estimated that 75% of telemedicine initiatives fail during the operational phase [8], with most ventures failing to scale beyond their pilot stage [9]. Understanding key aspects that contribute to the overall success of telemedicine initiatives is crucial to allow telemedicine to reach its full potential [10].

In an analysis of 221 studies of telehealth interventions, Granja *et al.* [11] defined 27 categories for reasons that such interventions can fail. Quality of healthcare was the most significant contributing factor to the success of interventions, while cost was the most prominent factor contributing to failure [11]. Additional failure modes include the lack of involvement of stakeholders, reliability of equipment, health professionals' opinions and the perceived need for telehealth [12]. In another analysis of 35 entrepreneurial health ventures and 17 publications on telemedicine and mobile health (mHealth) projects, the significant reasons for failure were: mismatch between the solution and ecosystem, for example, limited access to technology and electricity in the target geography and incompatibility of the solution with local gender and cultural dynamics; inadequate training of providers; mistrust among the patient population and insufficient revenue to implement and sustain the project [13]. Of these various failure modes, implementers could avoid the majority by using a thoughtful, thorough, and disciplined design process upfront. By eliciting stakeholder feedback early and frequently and rapidly iterating on the program design in response, practitioners can avoid the many potholes on the road to a successful telehealth project implementation [14].

Existing telemedicine frameworks call out the need for a multiphase approach to telemedicine: one comprising data collection, needs assessment, accessibility, perception and affordability, with a second phase balancing the demand for health services with an implementation plan that meets the needs of the intended population [15]. Others call for strategic planning of telemedicine interventions that include needs assessment and analysis, strategies for business, marketing, communication, detailed implementation plans and the development of criteria for evaluation [16]. A framework by Broens *et al.* [17] highlights the determinants of successful telemedicine interventions as technology, acceptance, financing, organization and policy. The Model for Assessment of Telemedicine Applications highlights how one may evaluate telemedicine interventions to determine their level of success. However, it does not offer guidance in the design of the telemedicine program [18]. Another paper used the Osterwalder Business model canvas to review existing telemedicine business models [19]. Other program planning frameworks used in digital health, such as ‘Planning an Information Systems Project: A Toolkit for Public Health Managers’ by the World Health Organization and PATH [20] and ‘The mHealth Planning Guide: Key Considerations for Integrating Mobile Technology into Health Programs’ by USAID [21] provide resources for public health program managers to understand key aspects of implementing digital health programs, but are not specific to the implementation of telemedicine.

These frameworks are helpful and call out some critical aspects of telemedicine program design; however, they are not explicitly built to aid implementers in project design and planning. Without an active and intentional effort to the contrary, those involved in project design end up in isolated silos. Doctors tend to focus on the clinical aspects, engineers on software and hardware, managers on funding and little or no cross-fertilization of ideas [22]. This paradigm is not conducive to successful telemedicine design, as it is critical that all of the individual components are synergistic and conceived as part of a comprehensive whole. Furthermore, interdisciplinarity has been demonstrated to be crucial for project success in business, healthcare, technology and research [23–25]. A framework is thus needed to systematize and operationalize the comprehensive design of early-stage telemedicine programs by all team members and overcome organizational and individual tendencies to isolate expertise.

## OBJECTIVES

Here, we propose a framework for designing telemedicine interventions in which all the necessary factors for the success of a project can be considered, visually communicated, iterated upon and evaluated. The framework, titled the Telemedicine Program Design Canvas, is a concise yet comprehensive tool for rapid prototyping that includes a systems-level view of the critical elements required to thoroughly and intentionally design successful design telemedicine programs.

## METHODS

We developed the Telemedicine Program Design Canvas through workshops with stakeholders and users of telemedicine. We conducted six workshops with various implementing organizations in five LMICs globally. We conducted five workshops in person and one virtually due to restrictions introduced by the COVID-19 pandemic. Each workshop was 16 to 20 hours long. Three projects were with non-profit organizations and three with government organizations. The workshops were conducted by a trained facilitator (authors VB or NV) and covered various topics to plan the telemedicine implementation. The facilitator guided the participants by developing a theory of change and an implementation model for their specific telemedicine intervention. A notetaker took notes during the workshop. Participants used whiteboards and post-its for brainstorming. The primary language of the workshops was English, and the local language with translators present at each workshop as needed. A document with the project implementation plan was prepared at the end of every workshop.

The authors of this paper analyzed the materials from each workshop and the project implementation plans. In addition, five out of the six projects moved forward into the implementation stage. The authors reviewed project progress reports of these projects and identified deviations from the project implementation plan. Since the projects were implemented in various geographies and healthcare areas, the authors considered the transferability of intervention components and the relationship to the country and implementation context. The authors discussed these insights over several meetings conducted both in-person and online. The insights from these design workshops led to the Telemedicine Program Design Canvas development.

To test the Canvas, we applied it to the design and planning of six new telemedicine interventions with seven implementing organizations between 2020–21. Five were NGO projects, and one was a government project. Since the COVID-19 pandemic was at its peak during this period, the projects were primarily focused on mitigating the effects of the pandemic. We conducted these workshops online via videoconferencing due to travel restrictions. Through inputs from the collaborators in the testing and validation phase, we modified the canvas to make it as user-friendly and as widely applicable as possible by redesigning various aspects or adding more elements.

## RESULTS AND DISCUSSION

We present a combined results and discussion section that shares the planning canvas developed through these workshops and discuss the individual components of the canvas. A total of 12 telemedicine case studies and perspectives of 101 stakeholders from various health organizations contributed to the overall design of the telemedicine canvas. A diverse mix of stakeholders, including public health program managers/organizational leadership, healthcare providers including doctors, nurses, community health workers, engineers/technology staff and business leaders, participated in the workshops. In the first phase, we conducted six workshops that led to the development of the canvas. In total, 69 key stakeholders of telemedicine programs attended the workshops. Four workshops were conducted in the second phase for testing and validation of the canvas with a multidisciplinary group of stakeholders. These workshops were attended by 39 participants.

**Figure 1:**
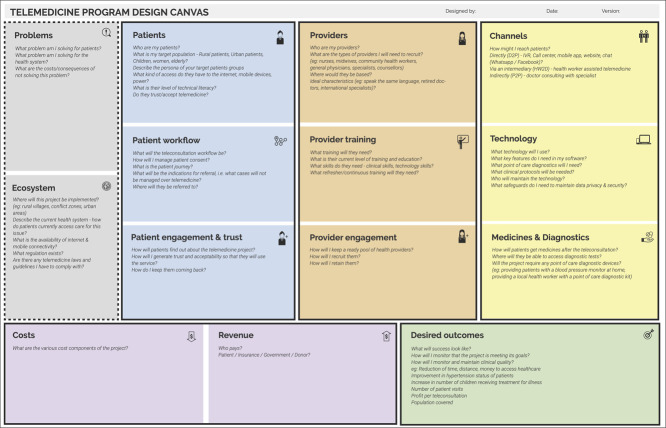
The telemedicine program design canvas.

We identified 14 key domains across six broad categories that project implementers must consider for successful telemedicine program execution. The final canvas is depicted in Fig. 1.

Visually, the Telemedicine Program Design canvas consists of 14 boxes (described in detail in Fig. 1). Implementers may fill out the canvas in any order. Within each box, example questions to be answered are indicated. These are merely intended to be a starting point for the discussion and may not be relevant to all interventions. The first section (gray) is the project background—namely, the problems to be solved and the program's environment. Telemedicine interventions are multisided market models connecting patients with providers. The second section (blue) addresses demand-side factors—the target patient population, the patient workflow, patient engagement and trust. Supply-side factors (orange) come next, including providers, provider training and provider engagement. The following section (yellow) includes the tools needed in the program—the channels that may connect patients and providers, technology, medicines and diagnostics required. The following sections go through costs and revenues (purple), followed by the project's desired outcomes (green).

### Problems

This section describes the pain points that the telemedicine program seeks to solve, such as reducing geographic access barriers, inequitable distribution of healthcare providers, improving support and training for frontline health personnel, increasing convenience for patients, reducing costs, improving health system efficiency, provide care during off-hours, provide continuous monitoring at home for chronic patients and reducing exposure to infections in the context of the COVID-19 pandemic. It is essential to consider the problems and the target beneficiaries. Does the program address pain points experienced by patients, providers, health systems or, as is often the case, all three? What are the consequences and costs associated with this problem not being solved for these stakeholders? If the issues to be solved are well and thoroughly described, it will be easy to define the value of your telemedicine program to each stakeholder. The range of healthcare problems that the implementing organizations want to solve can be diverse, including maternal health, child health, sexual reproductive health, elderly care, mental health, demonstrating the applicability of telemedicine to a range of medical specialties at the primary, secondary and tertiary care levels.

### Ecosystem

It is crucial to describe the context where the project will be implemented in terms of location and critical characteristics of the setting (for example, urban vs. rural). Your intervention must consider the existing systems and regulations in place, how the current health system works, how this problem is currently solved and what laws related to telemedicine, digital health or data privacy you need to consider. It is also essential to describe the status of the internet and mobile connectivity in the region to provide more background on what your potential solution can utilize.

### Patients

Describe who your solution will be targeting as the patient population and define the characteristics of the target patient group. Define their level of experience with, and acceptance of technology and telemedicine as these will shape your choice of channels and technology.

### Patient journey

Consider the individual parts of your intervention and how they relate to each other to define how your intervention will work. It is also valuable to examine this from a patient's viewpoint to describe precisely how they would move through your intervention and the steps they would take. Aim to answer important aspects of the patient journey, such as how they would learn about the project, connect with a provider immediately after initiating a virtual consultation request or book an appointment, and how patient consent would be recorded. There must also be a mechanism in place to make the proper referrals if a patient's condition is outside the scope of the telemedicine program, either due to urgency or complexity.

### Patient engagement and trust

Patient adoption and acceptability are vital to the success of an intervention and often the most challenging aspect of the intervention. You should iterate upon how you plan to make patients aware of your intervention and generate the trust required for them to utilize it. Accordingly, projects came up with different strategies for patient outreach, such as posters, pamphlets, murals, advertisements on radio, television and newspapers, marketing on social media, door-to-door flyer distribution, word of mouth marketing by local health workers, volunteers or key opinion leader marketing, outbound marketing calls to name a few. Each method has associated costs, reach, scalability and effectiveness tradeoffs, so it is important to consider multiple options and deliberate as part of the program and business sustainability design.

### Providers

Different types of healthcare providers may participate in telemedicine-based care delivery, resulting in clinical practice implications. These may include specialist doctors, general physicians, community health workers, nurse midwives and other healthcare professionals. The level of training and skill of the health provider impacts the range of services offered to patients and the cost of the intervention. Various models for recruitment of remote physicians across the projects that participated in the design workshops included using pro-bono volunteer doctors, paid full-time virtual doctors, identifying doctors already on the payroll of the health organization and creating a blended schedule of in-person and virtual clinic days, working with senior retired physicians or medical students with low bono stipends or using paramedical staff, such as nurses and counselors to handle patient cases.

### Provider training

You need to consider who needs to be trained, what their starting level of training is, what skills they need to obtain and what kind of continuous training they will need to undergo. Training may include but is not limited to the use of technology, ‘web-side manner’, soft skills, care practice guidelines for telemedicine, legal and ethical norms, data privacy and the limitations of telemedicine.

### Provider engagement

Like patient engagement, you need to maintain a ready pool of health providers to deliver your intervention. You must consider how you will find them and get them involved in your program and how you can keep them engaged as the project continues. Addressing provider acceptance and adoption is essential to the success of the intervention. We found this to be especially important in government projects where motivation levels of health providers may be low, where they may be overburdened, or may be reluctant to adopt new technology.

### Channels

A direct-to-patient approach connects a patient with a provider. In contrast, a provider-to-provider approach connects providers with a lesser skill level to those with a higher skill level. Channels may include app-based or web-based video consultations, phone helplines or chatbots. The interactions may be synchronous (in real-time) or asynchronous (store-and-forward). The choice of channel largely depends on the earlier sections of the canvas.

**Figure 2:**
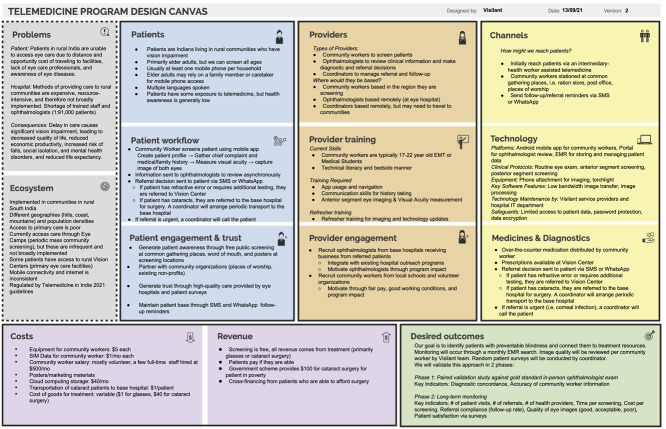
A completed telemedicine program design canvas for Visilant—delivering eye care to rural India through Teleophthalmology, a project by Johns Hopkins Center for Bioengineering Innovation & Design, Global Institute for Vision Equity, & Aravind Eye Care.

### Technology

Technology is at the core of a telemedicine program. This includes the software and hardware that patients and providers use to interact and transmit information and any diagnostic tools and protocols. In this section, it is critical to ensure that the chosen technology is compatible with the ecosystem in which the program will be operating. For example, in a location with unreliable internet, the technology should function offline, perhaps with store and forward functionality. Implementers should also consider infrastructure for tech support, maintenance, ease of use, cost, adaptability, interoperability, security and use of appropriate standards.

**Figure 3:**
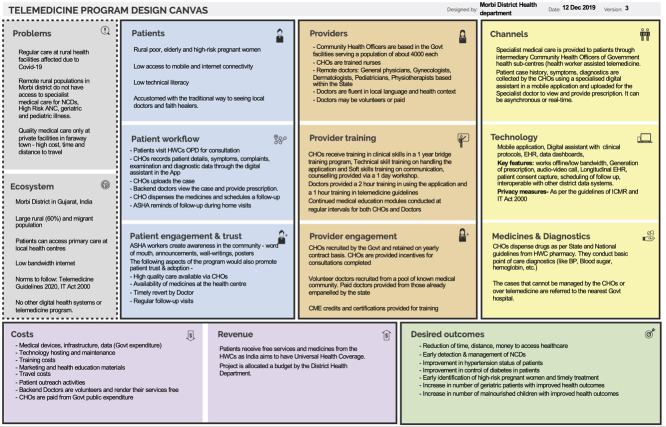
A completed telemedicine program design canvas for MyTeleDoc, a project by the District Health Department of Morbi.

### Medicines and Diagnostics

Beyond diagnosis, to achieve the desired outcome of improved health, many telemedicine programs will need to provide a pathway for patients to access tests and medicines. Therefore, the program designers must work out where, when and who will supply medicines and how they will be prescribed and paid for. If patients need to conduct additional diagnostic tests, the project designers must identify if the tests can be conducted via point of care diagnostic devices or referrals to other facilities.

### Costs

In this box, designers should consider elements, such as capital and operational expenses, that fall into the various categories involved in a program, including technology, supplies, upfront training fees and more. While telemedicine programs may reduce costs for patients, they may increase costs for health organizations due to additional technology, training and staffing.

### Revenue

Here, the user describes all revenue streams that will offset the costs outlined in the previous section. This should include who is funding the project and how and their motivations for doing so. For example, patients may pay out-of-pocket because the telehealth program provides a desirable service, while non-governmental organizations may fund a project through grants because it aligns with their stated mission. Depending on the healthcare ecosystem, third-party payers, such as insurance companies, the government and employers may also be part of the revenue structure, in which case implementers must account for their purviews and policies.

### Desired outcomes

Clear goal setting provides orientation for the entire telehealth project. You must clearly state what a successful project would achieve, and these should be aligned with the goals of the funders identified in the previous section. Expected outcomes for telemedicine projects include reduced cost and distance traveled for patients, improved convenience and health-seeking behaviors, reduced costs for the health system and improved healthcare outcomes or comparable outcomes to face-to-face care at lower prices. Telemedicine can be used to increase the reach of a healthcare facility well beyond its geographical limitations. Reach is necessary to justify costs invested in the project. Defining these success metrics at the outset of a project and regularly monitoring them throughout implementation allows the program to be agile and resource-efficient by rapidly identifying and correcting issues as they arise.

Furthermore, these key indicators—objective evidence that your project is effective in its stated purpose—comprise the data you will present to future funders to justify your work. Of note—and a step that is often overlooked—is that reliable collection of these data should be deeply ingrained in your project’s processes, technology and infrastructure. For a program to be effective, the measurement of intervention success cannot be an afterthought in its design.

## CASE STUDIES

### Case study: Visilant—Increasing access to eye care in rural India through teleophthalmology

This project uses a community-based, volunteer-driven care model to enable broad, cost-effective and ongoing eye screening in rural India. Volunteers are guided through medical history taking, a physical examination of the eye, measuring visual acuity and capturing clear anterior segment images using a smartphone application called Intelehealth [26]. A remote ophthalmologist reviews this information. Patients with pathologies can be prescribed treatment, referred to a rural vision center for further testing or transported to a base hospital for free or subsidized cataract surgery. Fig. 2 shows a completed Telemedicine Canvas for this project.

### Case study: MyTeleDoc

The project connects nurses, called community health officers, in government-run rural primary care clinics called Health and Wellness Centers, with specialists at secondary and tertiary health facilities. The project was implemented in the Morbi district of Gujarat, India. The goal of the program was to provide care for patients who are typically referred, such as women with high-risk pregnancies, children with severe malnourishment, patients with musculoskeletal issues, dermatology issues. Fig. 3 shows a completed Telemedicine Canvas for this project.

## LIMITATIONS

While the canvas was designed through a case-study-based approach using 12 telemedicine interventions, a greater number of interventions would be needed to prove the efficacy of this approach. We cannot show whether using the telemedicine program design canvas would in a more successful intervention. Anecdotally, our group believes that this canvas proves to be a valuable tool for the rapid prototyping and iteration of telemedicine programs. The case studies included direct-to-patient and provider-to-provider telemedicine but did not include other types of telemedicine, like telesurgery and telementoring. The canvas can help stakeholders from different backgrounds to collaborate and address the factors that contribute to a successful intervention. Still, our methods may not include the voices of each stakeholder equally, especially those of patients.

## CONCLUSIONS

The Telemedicine Design Canvas allows for comprehensive visualization of the critical factors that implementers should consider to implement a telehealth intervention successfully. This framework provides an organized method for evaluating and iterating upon the essential aspects of an intervention, as were defined by experiences with past telemedicine program implementations, and visually communicating the program design to team members and stakeholders. There are notable similarities and differences in how different implementing organizations approach a telehealth program based on context. By rigorously approaching the design process, organizations can mitigate the risk of downstream failure. We then proved the relevance of this canvas through the development and application of the canvas to 11 telemedicine interventions that this group has implemented. The Telemedicine Canvas was helpful in rapid prototyping of programs and considering various strategies to address the 15 key elements. The Canvas also serves as a communication tool to align internal and external stakeholders while considering diverse viewpoints.

Taken together, the high failure rate of telemedicine programs [8] and the increased use of telemedicine in LMICs indicates that there is a gap between the design and implementation of telehealth interventions that need to be resolved swiftly. Here, we present the Telemedicine Program Design Canvas—a visual framework for brainstorming, iteration and collaboration on telehealth projects. The Telemedicine Program Design Canvas contributes to narrowing this gap by providing a robust framework for the holistic, intentional and comprehensive design of telemedicine interventions that facilitates collaboration and rapid iteration. Through participatory design workshops, implementer experiences and application of the Canvas to multiple successful global telehealth projects, we have identified 14 key domains that implementers must consider during the early-phase design process. Incorporating this tool as part of a thorough, iterative design process may enable organizations to avoid the pitfalls that cause telemedicine programs to fail during the operational phase.

## ACKNOWLEDGEMENTS

We would like to thank Dr. Bimal Buch, Dr. Hardik Rangparia and Dr. JM Katira for their support in the design of the MyTeleDoc project used as a case study in this paper. We would also like to thank the following organizations and their representatives for participating in the design workshops for their telemedicine projects—Arogya Foundation of India & Ekal Abhiyan, Syrian American Medical Society, District Health Department Morbi, District Health Administration of Mindanao Phillipines, VSO International, Ministry of Health of the Kyrgyz Republic, UNICEF Kyrgyzstan, Aaroogya Foundation, National Health Mission Jharkhand, Transform Rural India, Uninhibited, Swami Vivekanand Health Mission, Medicines Sans Frontiers (MSF) India, Ibis Reproductive Health, Vikalp Sansthan, Johns Hopkins University Center for Bioengineering Innovation and Design, Aravind Eye Hospital and Global Institute for Vision Equity.

All persons who have made substantial contributions to the work reported in the manuscript (e.g., technical help, writing and editing assistance, general support), but who do not meet the criteria for authorship, are named in the Acknowledgements and have given us their written permission to be named. If we have not included an Acknowledgements, then that indicates that we have not received substantial contributions from non-authors.

## FUNDING SOURCE

None.

## CONFLICT OF INTEREST STATEMENT

Neha Verma is a founder of and serves as the CEO of Intelehealth, a 501(c) [3] non-profit supporting the development and implementation of the telemedicine software. Soumyadipta Acharya is a founder of and serves as the Board President of Intelehealth. Neha Verma and Soumyadipta Acharya are also the inventors of the technology involved in the Intelehealth app, which was used in the study described in this publication. This arrangement has been reviewed and approved by the Johns Hopkins University in accordance with its conflict of interest policies.

## AUTHORSHIP STATEMENT

Manuscript title: The Telemedicine Program Design Canvas: a visual tool for planning telemedicine interventions.

Neha Verma^1^, Izabella Samuel^2^, Samuel Weinreb^2^, Mackenzie Hall^2^, Kai Zhang^2^, Mariana Bendavit^2^, Vibha Bhirud^3^, Jordan Shuff^2^, Youseph Yazdi^2^ and Soumyadipta Acharya^2^.

All persons who meet authorship criteria are listed as authors, and all authors certify that they have participated sufficiently in the work to take public responsibility for the content, including participation in the concept, design, analysis, writing or revision of the manuscript. Furthermore, each author certifies that this material or similar material has not been and will not be submitted to or published in any other publication before its appearance in the Lancet Digital Health.

## AUTHORSHIP CONTRIBUTIONS

*Category 1.*

Conception and design of study: N. Verma, V. Bhirud, S. Acharya, Y. Yazdi.

Acquisition of data: N. Verma, V. Bhirud.

Analysis and/or interpretation of data: N. Verma, V. Bhirud, I. Samuel, S. Weinreb, M. Hall, K. Zhang, M. Bendavit, J. Shuff.

*Category 2.*

Drafting the manuscript: N. Verma, I. Samuel, S. Weinreb, M. Hall, K. Zhang, M. Bendavit, J. Shuff, S. Acharya.

Revising the manuscript critically for important intellectual content: N. Verma, I. Samuel, S. Weinreb, M. Hall, K. Zhang, M. Bendavit, J. Shuff, S. Acharya, V. Bhirud, Y.Yazdi.

*Category 3.*

Approval of the version of the manuscript to be published (the names of all authors must be listed): N. Verma, I. Samuel, M. Hall, K. Zhang, M. Bendavit, V. Bhirud, J. Shuff, Y. Yazdi, S. Acharya.

We would like to note the untimely death of S. Weinreb under tragic circumstances due to which he has not been listed in Category 3. S. Weinreb participated extensively in the writing of this manuscript and would have been listed as a co-author without any doubt. We request the editors to consider his inclusion in the list of co-authors and request guidance on how to proceed.

## DATA AVAILABILITY STATEMENT

The data underlying this article are available in the article*.*
